# Development of New IL-1R Antagonists with Improved Anti-inflammatory Efficacy

**DOI:** 10.7150/thno.120259

**Published:** 2026-01-01

**Authors:** Mooseok Kang, Ae-Ree Lee, Hyeji Jung, Gyubin Jang, Byeongchan Kim, Sung-Hyun Yoon, Je-Wook Yu, Jaewon Ko, Ji Won Um, Iksoo Chang

**Affiliations:** 1 Cytokine Innovation Center, iProtein Therapeutics Co. Ltd., Industry-University Cooperation Building R7-208, 333 Techno Jungangdae-Ro, Hyeonpoong-Eup, Dalseong-Gun, Daegu 42988, Korea.; 2 Department of Brain Sciences, Daegu Gyeongbook Institute of Science and Technology (DGIST), 333 Techno Jungangdae-Ro, Hyeonpoong-Eup, Dalseong-Gun, Daegu 42988, Korea.; 3 Center for Synapse Diversity and Specificity, Daegu Gyeongbook Institute of Science and Technology (DGIST), 333 Techno Jungangdae-Ro, Hyeonpoong-Eup, Dalseong-Gun, Daegu 42988, Korea.; 4 Department of Microbiology and Immunology, Institute for Immunology and Immunological Diseases, Brain Korea 21 PLUS Project for Medical Science, Yonsei University College of Medicine, Seoul 03722, Korea.

**Keywords:** inflammation, anti-inflammatory efficacy, interleukin-1, interleukin-1 receptor, Interleukin-1β, interleukin-6, anakinra, antagonist, in silico protein design, molecular dynamics simulations, thermodynamic integration, binding free energy

## Abstract

**Background:** Anakinra, a recombinant human interleukin-1 receptor antagonist (hIL-1Ra), is a widely used anti-inflammatory biologic for conditions like rheumatoid arthritis and gout. However, its limited potency and dose-dependent side effects restrict broader therapeutic application, highlighting a need for more potent and stable IL-1R antagonists.

**Methods:** To develop improved IL-1R antagonists, we rationally designed six hIL-1Ra variants using structure-guided mutagenesis. Molecular dynamics simulations and thermodynamic integration predicted enhanced binding stability, with an average binding free energy improvement of -7.8 ± 0.9 kcal/mol compared to wild-type hIL-1Ra (hIL-1Ra WT). We assessed variant functions in microglia-derived HMC-3 cells by measuring IL-1β and IL-6 mRNA suppression and evaluated their ability to attenuate IL-1β-induced NMDAR hyperactivation in cultured cortical neurons using electrophysiological recordings. *In vivo* validation was performed using *Nlrp3*^D301N^ knock-in mice, a model of chronic neuroinflammation.

**Results:** All six hIL-1Ra variants demonstrated enhanced anti-inflammatory activity, suppressing IL-1β and IL-6 expression by 25-53% in HMC-3 cells. The E127Q variant exhibited the greatest efficacy. In primary cultured neurons, hIL-1Ra E127Q more effectively inhibited IL-1β-induced NMDAR-mediated postsynaptic responses at lower concentrations than hIL-1Ra WT. Furthermore, acute administration of hIL-1Ra E127Q, but not hIL-1Ra WT, reversed elevated NMDAR activity in the medial prefrontal cortex of *Nlrp3*^D301N^ knock-in mice.

**Conclusion:** This study successfully developed next-generation hIL-1Ra variants with superior receptor binding and anti-inflammatory activity. E127Q emerged as a promising therapeutic candidate, effectively attenuating inflammatory signaling and neuroinflammatory responses both *in vitro* and *in vivo*. These findings underscore the significant therapeutic potential of engineered IL-1R antagonists for treating inflammation-driven neurological and systemic disorders, paving the way for improved anti-inflammatory therapies.

## Introduction

Chronic inflammatory processes have been linked to a wide spectrum of conditions, including autoimmune disorders, cardiovascular diseases, and cancer 1, 2. During an inflammatory reaction, immune cells release cytokines and other mediators that orchestrate defense and healing processes. However, persistent production of pro-inflammatory cytokines can lead to tissue damage and contribute to disease pathogenesis 1. For example, excessive or prolonged inflammation underlies the pathology of rheumatoid arthritis, gout, and autoinflammatory syndromes, among others 2.

Among the key mediators of inflammation is interleukin-1 (IL-1), a pro-inflammatory cytokine family comprising subtypes IL-1α and IL-1β that plays a central role in initiating and sustaining immune responses 3, 4. Binding of IL-1 to the type I IL-1 receptor (IL-1R1) on target cells triggers recruitment of the IL-1 receptor accessory protein and activation of downstream signaling cascades, including NF-κB (nuclear factor-κB) and MAPK (mitogen-activated protein kinase) pathways, leading to induction of other inflammatory cytokines, chemokines, and enzymes that amplify the inflammatory response 4. IL-1-driven signaling is crucial in host defense but, left unchecked, it contributes to the development of acute and chronic inflammatory diseases 3, 4. To prevent excessive IL-1 activity, the body produces hIL-1Ra (human IL-1 receptor antagonist), a naturally occurring inhibitor of IL-1 that binds to IL-1R1 with high affinity but does not induce receptor signaling, thereby competitively blocking IL-1 from binding and activating the receptor 5, 6. This endogenous antagonist is pivotal in maintaining the balance of the IL-1-IL-1Ra axis and restraining inflammation under normal physiological conditions 5. An imbalance favoring IL-1 over IL-1Ra (such as in IL-1Ra-deficient mice or in certain autoinflammatory diseases) results in unchecked inflammation and tissue damage, underscoring the role of IL-1Ra as a crucial negative regulator of IL-1-mediated inflammation 6.

The importance of IL-1 in disease has motivated the development of therapeutic IL-1 blockers. One such blocker is anakinra, a recombinant, non-glycosylated form of hIL-1Ra with an N-terminal methionine that was approved for clinical use in 2001 for rheumatoid arthritis (RA) and has since been used in other inflammatory conditions, such as cryopyrin-associated periodic syndromes (CAPS) and systemic idiopathic juvenile arthritis 7, 8. By competitively inhibiting IL-1 signaling, anakinra reduces inflammation and ameliorates disease symptoms. In RA, for instance, anakinra treatment leads to reduced joint swelling and pain, and in CAPS (autoinflammatory syndromes driven by excessive IL-1β), it can dramatically quell disease flares 7, 8. In head-to-head comparisons with other cytokine inhibitors (e.g., anti-TNF therapies), anakinra has shown relatively modest efficacy in RA, highlighting an unmet need for more potent and/or longer-acting IL-1-targeting agents 8, 9. These limitations have spurred the development of next-generation IL-1 inhibitors with improved dosing convenience and effectiveness, such as the IL-1β monoclonal antibody canakinumab and the IL-1 Trap Rilonacept 10, 11. Nonetheless, its unique mechanism and broad IL-1 blocking ability make anakinra an attractive starting point for engineering improved “bio-better” therapeutics.

Given these limitations, there is a strong rationale for developing enhanced hIL-1Ra variants with superior anti-inflammatory performance. The goals of efforts to develop bio-better hIL-1Ra mutants include increasing their binding affinity for IL-1R1 (to more effectively outcompete IL-1) and maintaining or improving their safety. Prior attempts provide proof-of-concept that the therapeutic properties of hIL-1Ra can be improved. For example, directed evolution and mutagenesis approaches have yielded hIL-1Ra mutants with substantially higher antagonistic activity *in vitro* and greater efficacy *in vivo* than wild-type hIL-1Ra (hIL-1Ra WT) 12. These findings demonstrate that modifying the sequence of IL-1Ra leads to markedly boosted functions as an IL-1 inhibitor.

As a complement to experimental mutagenesis, *in silico* and structure-based design techniques now allow rational engineering of cytokine antagonists 13. Computational modeling of IL-1-IL-1Ra-IL-1R1 interactions can identify key binding interfaces and “hotspot” residues where mutations might strengthen receptor binding or stability of the ligand-receptor complex. Advances in protein modeling, docking simulations, and binding free energy calculations enable hIL-1Ra variants to be screened *in silico*, enabling prediction of variants with improved receptor affinity or pharmacological properties before validation in the laboratory. These capabilities provide a clear impetus for using computational approaches to guide the development of hIL-1Ra mutants. By leveraging structure-based design tools, we aim to develop bio-better hIL-1Ra that overcomes the limitations of current therapies and offers improved outcomes for patients with inflammatory diseases 14, 15. In this study, we employed a structure-based *in silico* strategy to identify hIL-1Ra variants with improved IL-1R1 binding stability, laying the groundwork for the development of next-generation anti-inflammatory therapeutics.

## Materials and Methods

### Supercomputing molecular dynamics simulation of IL-1R1-IL-1Ra complexes

To design mutant protein variants of hIL-1Ra with enhanced antagonistic activity towards IL-1R1, we used the IL-1R1-hIL-1Ra complex structure, registered in the Protein Data Bank (PDB ID: 1IRA) 16, as a framework. Molecular dynamic (MD) simulations of the protein were performed using the AMBER package 17, 18. The aqueous *in vivo* environment was simulated by surrounding the initial structure of IL-1R1-IL-1Ra used in structural simulations with virtual water, prepared by solvating the complex structure in an octahedron-shaped TIP3P (transferable intermolecular potential with 3 points) “water box” 19 that extended 15 Å beyond the outermost atoms of the complex. Periodic boundary conditions were applied based on the water box, and the system was neutralized by adding Na^+^ and Cl^-^ ions to balance net charges. The stability of three-dimensional (3D) arrangements of amino acid side chains and water molecules constituting the IL-1R1-hIL-1Ra complex was ensured by constraining hydrogen bond lengths using the SHAKE algorithm and by performing energy minimization using the ff14SB force field 20, with long-range electrostatic interactions calculated with the Particle-mesh Ewald (PME) method (cutoff distance, 9 Å). The minimization protocol consisted of 2000 steps of steepest descent, followed by 2000 steps of conjugate gradient, resulting in a total of 4000 minimization steps. Following minimization, diverse conformational ensembles were sampled by conducting MD simulations, utilizing a 2 fs time-step, with application of the SHAKE algorithm to constrain hydrogen bonds, and PME for long-range electrostatics at a 9 Å cutoff. The system was heated from 100 K to 300 K under NVT (number, volume, temperature) ensemble conditions for 100 ps with a positional restraint of 15 kcal/mol. Equilibration was subsequently performed at 300 K under NPT (number, pressure, temperature) ensemble conditions for 5 ns. Production MD runs were conducted under NPT ensemble conditions at 300 K and 1 bar for 100 ns, and 10 independent trajectories were generated for each simulation condition. From each trajectory, 100 conformational ensembles were collected and analyzed. The CPPTRAJ toolset in the AmberTools18 package was used for the basic calculations 18, and all protein structure images were created using VMD software 21. Throughout the text, the IL-1Ra residue numbering reflects that of the recombinant human IL-1Ra sequence.

### Calculation of differences in binding free energy using the thermodynamic integration method

The thermodynamic integration (TI) method 22, 23 was employed to analyze binding free energy differences (ΔΔG), which reflects changes in binding stability between mutated hIL-1Ra variants and IL-1R1. Protein binding free energy was calculated using a thermodynamic cycle in combination with TI. ΔG1 represents the free energy difference between WT and mutant hIL-1Ra in the bound state, whereas ΔG3 represents the same free energy difference in the unbound state. ΔG2 represents the free energy difference between bound and unbound states of WT hIL-1Ra, whereas ΔG4 represents the binding free energy of the mutant hIL-1Ra. ΔG1 and ΔG3 were calculated using TI, and the change in free energy caused by the mutation (ΔΔG) was computed as ΔΔG = ΔG2 - ΔG4 = ΔG1 - ΔG3 by thermodynamic equivalence. Details of the formulations can be found in **Supplementary Methods**.

### Expression and purification of WT and mutant hIL-1Ra plasmids

Based on results of supercomputing MD simulations, we (iProtein Therapeutics (iPT)) produced eight hIL-1Ra mutants - E127N, E127S, E127K, E127Q, E127C, E127D, E127H and E127V - as well as wild-type hIL-1Ra (designated iPT hIL-1Ra WT). The amino acid sequences of the designed mutant hIL-1Ra proteins were converted into corresponding nucleotide sequences using the Sequence Manipulation Suite (SMS). Final DNA sequences for mutant proteins were determined by comparison with the nucleotide sequence of hIL-1Ra WT. Synthetic DNA fragments encoding each mutant protein were synthesized (Bioneer), amplified by polymerase chain reaction (PCR), and cloned into *Nde*I and *Xho*I restriction sites in the pET21a vector by ligation-independent cloning (LIC) using the EZ-Fusion HT Cloning Kit (Enzynomics). The constructed plasmids were transformed into *Escherichia coli* DH5α and, after amplification, were selected on LB media containing 100 µg/ml ampicillin at 37 °C. The integrity of plasmids was verified by comparing their sizes with that of the hIL-1Ra WT plasmid using electrophoresis. Verified plasmids were transformed into *E. coli* BL21(DE3) RIL for large-scale protein production. Cells were cultured at 37 °C in LB media containing 100 µg/ml ampicillin and 50 µg/ml chloramphenicol. After cultures reached an optical density at 600 nm (OD_600_) of 0.6-0.8, protein expression was induced by adding IPTG (isopropyl β-D-thiogalactopyranoside) to a final concentration of 0.5 mM, followed by further incubation for 4 h at 37 °C. Cells were harvested by centrifugation at 9,370 × g for 20 min at 4 °C and stored at -80 °C until further processing. Cells were resuspended in lysis buffer (50 mM Tris-HCl pH 7.5, 150 mM NaCl, 1 mM PMSF, 0.25% Tween-20) and lysed by sonication (Q700-Sonicator), with application of pulses of 3 s on and 10 s off for 6 min on ice. Lysates were clarified by centrifugation (11,800 × g, 30 min, 4 °C) and filtered through a 0.45-µm syringe filter. Ion exchange chromatography was performed using a HiTrap Q HP column (Cytiva) equilibrated with 20 mM Tris-HCl buffer (pH 7.5). Bound proteins were eluted using a linear gradient of NaCl (0 to 1 M). Fractions containing target proteins were identified by sodium dodecyl sulfate-polyacrylamide gel electrophoresis (SDS-PAGE), concentrated using an Amicon Ultra-15 Centrifugal Filter Unit (10,000 kDa), and further purified by size-exclusion chromatography using a HiLoad Superdex 75 16/600 column equilibrated with phosphate buffer (20 mM phosphate pH 7.4, 150 mM NaCl). Purity and concentration were assessed by SDS-PAGE and NanoDrop spectrophotometry. The intact mass of purified proteins was verified using MALDI-TOF mass spectrometry (UltrafleXtreme MALDI TOF/TOF; Bruker). The amino acid sequences of purified proteins were further confirmed by liquid chromatography-tandem mass spectrometry (LC-MS/MS) using a hybrid quadrupole-Orbitrap mass spectrometer (Q Exactive PLUS; Thermo Scientific) coupled with nano-LC (ACQUITY UPLC M-Class system; Waters).

### Secondary structure analysis of WT and mutant hIL-1Ra by circular dichroism

The secondary structures of WT and mutant hIL-1Ra proteins were verified by circular dichroism (CD) spectroscopy. Samples (0.2 mg/ml), in a 0.1 cm quartz cuvette, were analyzed using a JASCO-1500 spectrometer. Spectra were recorded from 190 to 250 nm at a scan speed of 20 nm/min, a bandwidth of 5 nm, and a digital integration time (D.I.T) of 4 s. Data obtained from five scans were averaged and baseline-corrected using buffer measurements. Results indicated that mutations did not significantly alter secondary structure compared with the hIL-1Ra WT protein.

### Binding affinity analysis of WT and mutant IL-1Ra by surface plasmon resonance (SPR) and microscale thermophoresis (MST)

Binding affinities of WT and mutant IL-1Ra to IL-1R1 were measured using SPR and MST. For SPR, IL-1R1 was captured on a CM5 chip via a His Capture Kit, and various concentrations of IL-1Ra variants and IL-1β were flowed as analytes. Data were analyzed using a BiacoreT200 control and the BIAevaluation software. For MST, fluorescently labeled IL-1R1 was titrated with serially diluted IL-1Ra variants and IL-1β. Measurements were performed on a Monolith NT.115-pico machine, and the data were analyzed using the MO affinity analysis program. Detailed experimental protocols are provided in the **Supplementary Methods**.

### Antibodies

The following commercially available antibodies were used: rabbit monoclonal anti-NF-κB p65 (clone D14E12; Cell Signaling Technology; Cat# 8242; RRID: AB_10859369), rabbit monoclonal anti-Phospho-NF-κB p65 (Ser536) (clone 93H1; Cell Signaling Technology; Cat# 3033; RRID: AB_331284), mouse monoclonal anti-IκBα (clone L35A5; Cell Signaling Technology Cat# 46001; RRID: AB_3697353), mouse monoclonal anti-β-actin (Santa Cruz; Cat# sc-47778; RRID: AB_626632), rat monoclonal anti-CD45 (BioLegend; Cat# 103116, RRID: AB_312981), rat monoclonal anti-CD11b (Thermo Fisher Scientific; Cat# 53-0112-82; RRID: AB_469901), rat monoclonal anti-B220 (BioLegend; Cat# 103208; RRID: AB_312993), rat monoclonal anti-CD3 (BioLegend; Cat# 100235; RRID: AB_2561455), and rat monoclonal anti-CD4 (BD Biosciences; Cat# 560468; RRID: AB_1645271).

### Animals

All animals were housed in the DGIST Laboratory Animal Resource Center under routine husbandry conditions, with unrestricted access to food and water. All experimental procedures involving mice were reviewed and approved by the DGIST Institutional Animal Care and Use Committee (IACUC; protocol DGIST-IACUC-25082202-0001) and were conducted in full compliance with institutional guidelines for rodent research. *Nlrp3*^D301N^ and *Cx3cr1*-CreERT2 mouse lines (JAX #017971 and #021160) were sourced from Jackson Laboratories. All experimental procedures were performed on 7~12-week-old mice. Pregnant rats were purchased from Daehan Biolink for the preparation of *in vitro* cultures of rat primary cortical neurons.

### Evaluation of the anti-inflammatory efficacy of WT and mutant hIL-1Ras on the inflammatory cytokine-secreting HMC-3 cell line

To evaluate the anti-inflammatory effects of WT and mutant hIL-1Ra, we used the HMC-3 (human microglia clone 3) cell line, which is derived from microglia that secrete inflammatory cytokines upon inflammatory stimulation. Cells were cultured in minimum essential medium (MEM) supplemented with 10% fetal bovine serum (FBS) and 1% penicillin/streptomycin (P/S) and maintained at 37 °C in a 5% CO_2_ incubator. The eight hIL-1Ra mutant variants and iPT hIL-1Ra WT, produced as described above, as well as one commercially available recombinant protein (R&D Systems), termed RD hIL-1Ra WT - all at a concentration of 25 ng/ml - were tested for their ability to block an inflammatory response induced in HMC-3 cells by interleukin-1β (IL-1β; PeproTech), used at a concentration of 1 ng/ml. HMC-3 cells were concurrently treated with IL-1β and WT or mutant hIL-1Ra proteins for 24 h. RNA was then extracted from treated cells using the TaKaRa MiniBEST Universal RNA Extraction Kit (Cat# 9767A) and reverse transcribed into cDNA using the PrimeScript 1st Strand cDNA Synthesis Kit (Cat# 6110A). Quantitative reverse transcription-PCR (qRT-PCR) was performed using specific primers for *Il1b* (forward: 5'-AGC TAC GAA TCT CCG ACC AC-3'; reverse: 5'-CGT TAT CCC ATG TGT CGA AGA A-3') and *Il6* (forward: 5'-ACT CAC CTC TTC AGA ACG AAT T-3'; reverse: 5'-CCA TCT TTG GAA GGT TCA GGT-3'). PCR was conducted on a StepOnePlus RT-PCR system (Thermo Fisher) using TB Green Premix Ex Taq (Takara). Expression levels of inflammatory cytokine mRNAs, used to evaluate the anti-inflammatory efficacy of WT and mutant hIL-1Ra proteins, were quantified using the ΔΔCt method.

### Evaluation of anti-inflammatory efficacy using a mouse model of chronic neuroinflammation

To induce microglial expression of the NLRP3 D301N variant, *Nlrp3*^D301N^ mice were crossed with *Cx3cr1*-Cre^ERT2^ animals, which express tamoxifen-inducible Cre recombinase under the *Cx3cr1* promoter. Experiments were conducted 4 weeks after tamoxifen (T5648; Sigma) dosing, a period sufficient for long-lived microglia to retain the induced mutation while peripheral monocytes were replaced by newly generated *Cx3cr1*⁺ cells carrying the wild-type *Nlrp3* allele. Cre-dependent knock-in was induced by giving tamoxifen dissolved in corn oil to 6-week-old mice, administered intraperitoneally (i.p.) at 100 mg/kg/day for five consecutive days. After a 4-week period to permit turnover of peripheral monocytes, mice were i.p.-injected with 0.5 mg/kg lipopolysaccharide (LPS; *E. coli* O111:B4, L3012, Lot# 12 170 308; Sigma) or phosphate-buffered saline (PBS) twice at a 24-h interval to potentiate neuroinflammation. At 6 days post-LPS injection (dpi), animals were i.p.-injected with recombinant hIL-1Ra (5 or 10 mg/kg) at 1 h prior to sacrifice for *ex vivo* slice recordings.

### Primary neuron cultures

Primary cortical neurons were prepared as previously described 24. At 13 days *in vitro* (DIV 13), cultured rat cortical neurons were treated with vehicle (DPBS) or IL-1β (1 or 10 ng/ml) for 24 h, with or without pretreatment with the indicated concentration of hIL-1Ra 10 min prior to the application of IL-1β.

### *Ex vivo* electrophysiological recordings

Whole-cell recordings were performed in acute mouse brain slices using procedures described previously 25.

### Whole-cell recordings in cultured neurons

Electrophysiological recordings from cultured cortical neurons were performed as previously described in our earlier study 25.

### Immunoblot analysis

To directly assess IL-1R pathway blocking efficiency of WT and E127Q IL-1Ra, HMC-3 cells were treated with IL-1β (10 ng/ml) for 20 min with or without pretreatment of WT or E127Q hIL-1Ra proteins (50 ng/ml) 10 min prior to IL-1β treatment. 20 min after IL-1β application, cells were rinsed with ice-cold PBS and lysed in lysis buffer (50 mM Tris (pH 7.4), 1.0% Triton X-100, 150 mM NaCl, 1 µg/ml aprotinin, 1 µg/ml leupeptin, 1 µg/ml pepstatin, 1 mM Na_3_VO_4_, and 0.2 mM PMSF). Following a 30-min incubation on ice, samples were centrifuged at 16,000 × g for 20 min to obtain the clarified supernatants, and protein levels were subsequently quantified using the Bradford method. Obtained cell lysates were loaded on the SDS pages in equal amounts and immunoblotted with anti-NF-κB p65, anti-phospho-NF-κB p65, anti-IκBα, and anti-β actin antibodies, as indicated in each figure. Western blot images were quantified using ImageJ software (National Institutes of Health).

### Evaluation of anti-inflammatory efficacy using IMQ-induced psoriasis mouse model

Adult C57BL/6J mice (older than 7-week-old) were topically treated with 30 mg of either Cetaphil or Aldara cream containing 5% imiquimod (IMQ) under anesthesia induced by 2% isoflurane for consecutive 6 days on each ear. Concurrently WT or E127Q hIL-1Ra were i.p.-injected at daily doses of 5 mg/kg and thickness of both ears were measured daily using digital caliper until 7th day. On 7th day, mice were sacrificed after last ear thickness measurement and both ears were dissected out, homogenized in 1 mL of Trizol reagent and processed for RNA extraction, cDNA synthesis and qRT-PCR as described above. PCR amplification was performed using the following gene-specific primer sets: mouse *Il17a*, 5'-ATC CCT CAA AGC TCA GCG TGT C-3' (forward) and 5'-GGG TCT TCA TTG CGG TGG AGA G-3' (reverse). Primer sequences for mouse *Il4* and *Gapdh* were previously described 25.

### Determination of hIL-1Ra concentration in blood serum

Adult C57BL/6J mice were received a single i.p. injection of 5 mg/kg WT or E127Q hIL-1Ra. 1, 2, 3, 6 and 24 h after hIL-1Ra administration, blood samples were collected via retro-orbital bleed under anesthesia by i.p. injection of 2% Avertin solution (2,2,2-tribromoethyl alcohol dissolved in Tert-amylalcohol (Sigma)) dissolved in saline. Blood samples were centrifuged at 2,000 × g at 4 °C for 20 min after coagulation to prepare serum. Serum concentrations of hIL-1Ra were determined by using the human IL-1Ra DuoSet ELISA kit (Cat# DY280; R&D Systems) according to the manufacturers protocol.

### Evaluation of immunogenicity using flow cytometry and qRT-PCR

Adult C57BL/6J mice were i.p.-injected with 5 mg/kg WT or E127Q hIL-1Ra or phosphate-buffered saline (PBS) for 3 days. 24 h after last i.p. injection, mice were euthanized and perfused with PBS, and the lymph node, spleen, and lung tissue were carefully excised. Each tissue was homogenized with cold PBS and passed through a 70-µm cell strainer. To remove red blood cells, the homogenates were treated with RBC lysis buffer, and the resulting single-cell suspension was collected and washed with PBS for flow cytometry analysis. The following fluorochrome-conjugated monoclonal antibodies were used for staining: anti-CD45, anti-CD11b, anti-B220, anti-CD3, and anti-CD4. The analyses were performed using a FACS Verse (BD Biosciences) and the FlowJo software (TreeStar). Tissue were processed for RNA extraction, cDNA synthesis and qRT-PCR as described above. The following target genes were amplified using the indicated primer pairs: mouse *Cd19*, 5'-AAT GCT TCA GAC GTC AGG GA-3' (forward) and 5'-TGC CAC AGT GAG ATC TTG GT-3' (reverse); mouse *Il-2ra*, 5'-ACA AGA ACG GCA CCA TCC TA-3' (forward) and 5'-GGT GCA TAG ACT GTG TTG GC -3' (reverse); mouse *Pcna*, 5'-GTG GAG CAA CTT GGA ATC CC-3' (forward) and 5'-GGT TAC CGC CTC CTC TTC TT-3' (reverse); mouse *Rn18s*, 5'-CGC GGT TCT ATT TTG TTG GT-3' (forward) and 5'-AGT CGG CAT CGT TTA TGG TC-3' (reverse).

### Data analysis and statistics

All quantitative results are reported as means ± standard error of the means (SEMs). Normality of each dataset was examined using the D'Agostino-Pearson omnibus test. Depending on the characteristics of the data, comparisons were performed using Kruskal-Wallis test, or standard one- or two-way ANOVA followed by Tukey's post hoc analysis. Statistical analyses and generation of bar plots were carried out using Prism 8.0 (GraphPad Software).

## Results

### *In silico* design of eight hIL-1Ra variants with improved IL-1R1 binding stability compared with hIL-1Ra WT

**Figure 1:**
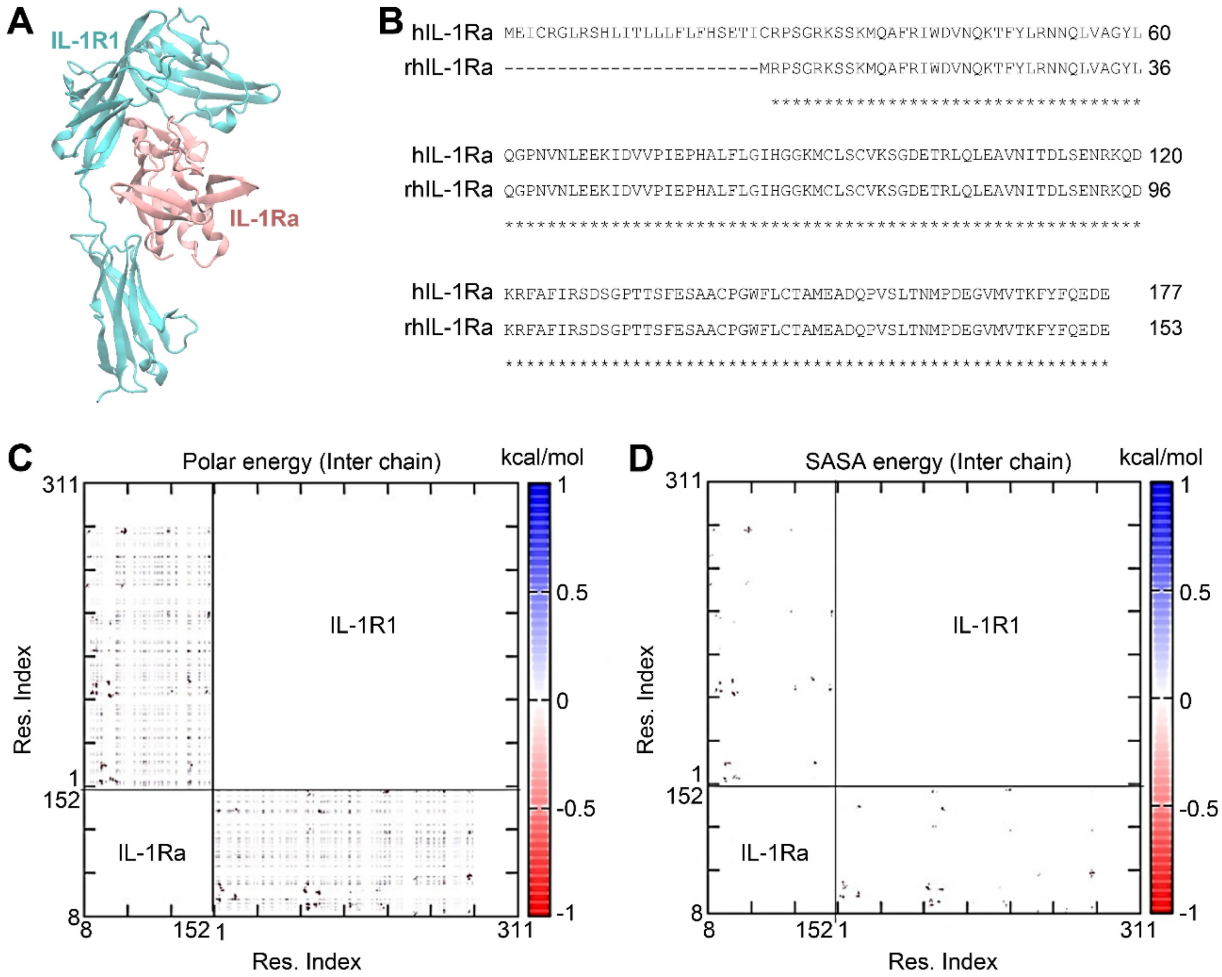
** IL-1R1-hIL-1Ra complex and pairwise interaction energy matrix of inter-chain amino acids in the complex.** (**A**) 3D structure of the IL-1R1 (cyan) and recombinant human IL-1Ra (rhIL-1Ra; pink) complex. (**B**) Sequence alignment of full-length IL-1Ra and rhIL-1Ra. (**C**) Polar energy, including contributions from electrostatic, van der Waals, and solvation energies. Residues of IL-1Ra are numbered according to the rhIL-1Ra sequence. (**D**) SASA energy [E (ΔSASAij)], calculated as the reduction in solvent-accessible surface area (ΔSASAij, in Å^2^) x surface tension (0.0072 kcal/mol·Å^2^). The IL-1Ra residue numbers are indicated above the sequences.

To design variants of hIL-1Ra that further improve the binding stability of hIL-1Ra to IL-1R1, we employed a structure-based *in silico* strategy. The structure of the hIL-1Ra-IL-1R1 complex (PDB ID: 1IRA) (**Figure 1A-B**) was equilibrated in an explicit water box and subjected to extensive MD simulations. From the sampled structural conformations of the IL-1R1-hIL-1Ra complex, we constructed pairwise interaction matrices of polar interaction energies (i.e., electrostatic + Van Der Waals + solvation energies [Eij]) and solvent-accessible surface area (SASA) energies [calculated as the reduction in SASA (ΔSASAij, in Å^2^) x surface tension (0.0072 kcal/mol·Å^2^), yielding ΔE(SASA_ij_)], for inter-amino acids (**Figure 1C-D**). Each pairwise interaction matrix was quantitatively analyzed according to network theory, resulting in 21 hub pairs of inter-amino acids, showing a polar energy ≤ -1 kcal/mol and a ΔSASAij ≥ 5 Å^2^ across the binding interface between hIL-1Ra and IL-1R1.

**Figure 2:**
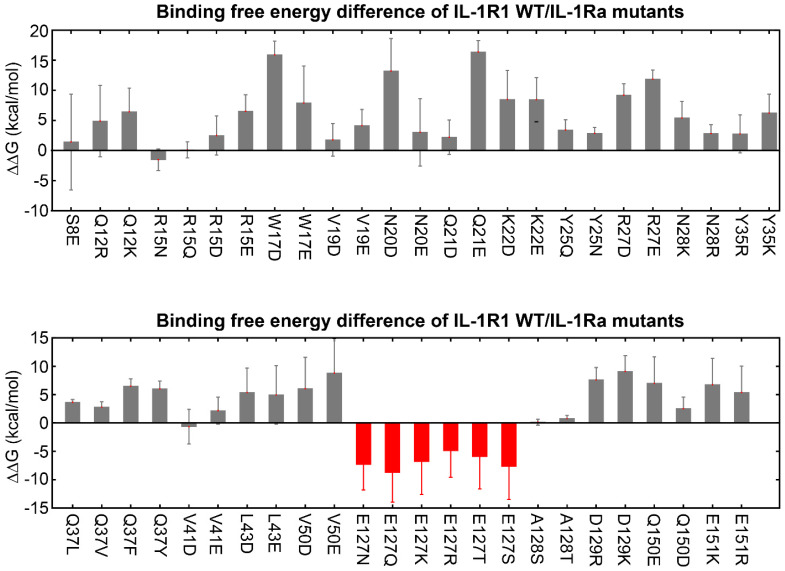
** Predicted binding free energy differences (ΔΔG) for single-point mutations at 21 identified hub amino acid positions in hIL-1Ra.** Changes in ΔΔG were calculated using thermodynamic integration-molecular dynamics (TI-MD) simulations. Mutations at the E127 position (indicated in red) resulted in significantly decreased ΔΔG values (E127N, E127Q, E127K, E127R, E127T, E127S), while mutations at other hub residues generally yielded increases in the ΔΔG values.

Based on this preliminary finding, we generated a list of possible hIL-1Ra variants designed to target each of the 21 key binding hub positions in hIL-1Ra. We then applied thermodynamic integration-molecular dynamics (TI-MD) simulation to calculate the relative binding free energy changes (ΔΔG) for each hIL-1Ra variant (**Figure S1**). Among the mutations, those at the Glu127 (E127) position of hIL-1Ra significantly improved binding stability, leveraged by the decrease in strong repulsive electrostatic interactions between E127 of hIL-1Ra and E146 of IL-1R1 (**Figure 2** and** 3A**). To ensure the reliability of these TI-MD calculations, we evaluated standard convergence metrics, including sampling smoothness and free-energy stabilization. The smooth profiles of ⟨∂H/∂λ⟩, with corresponding error estimates, and cumulative ΔΔG profiles, showing stable post-equilibration plateaus, together demonstrate robust and sufficiently converged TI calculations (**Figure S2** and **S3**).

Having identified E127 as the key residue in hIL-1Ra that has the most influence on binding stability upon mutation, we performed full residue-scanning mutagenesis together with TI-MD simulation at position E127, excluding glutamic acid (the native residue) and proline (due to its severe structural impact). Among the 18 single-point variants generated, eight - E127N, E127S, E127K, E127Q, E127C, E127D, E127H and E127V - displayed favorable ΔΔG values, indicating improved functional potency compared with hIL-1Ra (**Figure S4** and** 3B**). We then cloned and purified each of these variants for subsequent experimental validation. Although the ΔΔG improvement was lower for the E127D variant, it was chosen as a reference for experimental validation of the other seven variants because it only reduced the amino acid's side-chain length without altering its net charge.

### Compared to IL-1β, WT IL-1Ra and the designed mutants, except for E127D, have similarly stronger binding affinities to IL-1R1

We used SPR to measure the binding affinity of IL-1R1 to IL-1β and IL-1Ra WT and mutant proteins, and found that IL-1β and IL-1Ra WT and mutant proteins all bound to IL-1R1. The K_D_ values (indicating binding strength) were 5.8 nM for IL-1β, 264 pM for IL-1Ra, 281 pM for E127N, 248 pM for E127S, 410 pM for E127K, 283 pM for E127Q, 396 pM for E127C, 3.5 nM for E127D, 256 pM for E127H, and 258 pM for E127V. The binding of IL-1β was the weakest, and all of the mutated variants, except for E127D, had binding strengths similar to that of WT IL-1Ra (**Figure S5**).

We then used MST to measure the binding affinity of IL-1R1 to IL-1β and IL-1Ra WT and mutant proteins. Based on the observed changes in fluorescence intensity, reflecting changes in concentration, we observed that IL-1β and IL-1Ra WT and mutant proteins all bound to IL-1R1. The K_D_ values were 187 nM for IL-1β, 3.79 nM for IL-1Ra, 6.06 nM for E127N, 2.45 nM for E127S, 5.54 nM for E127K, 3.84 nM for E127Q, 7.52 nM for E127C, 40.64 nM for E127D, 4.59 nM for E127H, and 4.61 nM for E127V. Although the absolute values of K_D_ differed between the SPR and MST analyses, E127S consistently exhibited the highest binding affinity. In contrast, WT IL-1Ra and all mutants, except E127D, had similar binding affinities to IL-1R1, which were stronger than that of IL-1β to IL-1R1 (**Figure S6**).

### Six mutants of hIL-1Ra exhibited improved anti-inflammatory efficacy in reducing both IL-1β and IL-6 mRNA expression compared with hIL-1Ra WT, with E127Q exhibiting the highest efficacy

**Figure 4:**
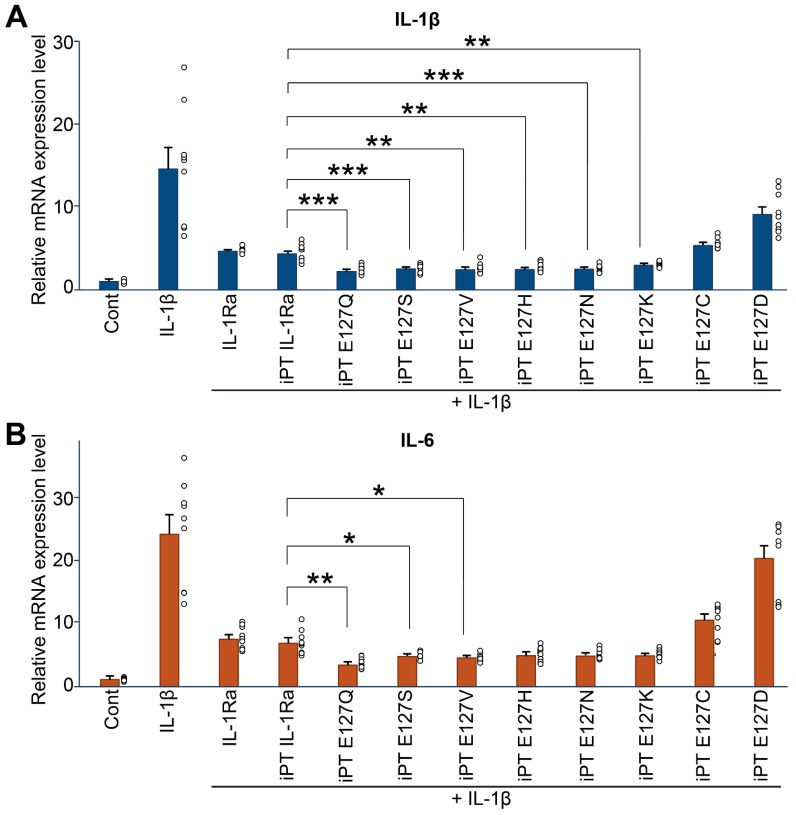
** Anti-inflammatory activity of WT and mutant hIL-1Ra variants.** (**A**) Inhibition of IL-1β expression. Unpaired two-tailed t-tests were performed between IL-1Ra WT and each mutant group. *p*-values were adjusted using the Bonferroni correction. Asterisks indicate significance based on adjusted *p*-values: **p < 0.01, ***p < 0.001. (**B**) Inhibition of IL-6 expression. Cont: untreated; IL-1β: IL-1β (1 ng/ml) only; IL-1β + RD hIL-1Ra: IL-1β (1 ng/ml) + R&D Systems hIL-1Ra WT (25 ng/ml); IL-1β + iPT hIL-1Ra WT: IL-1β (1 ng/ml) + iProtein Therapeutics hIL-1Ra WT (25 ng/ml); IL-1β + hIL-1Ra variants (E127N, E127S, E127K, E127Q, E127C, E127D, E127H, E127V): IL-1β (1 ng/ml) + mutant hIL-1Ras (25 ng/ml) produced by iPT. Statistical analysis was performed as described in (**A**). *p < 0.05, **p < 0.01.

We next evaluated mRNA expression levels of the pro-inflammatory cytokine, IL-1β, following treatment of HMC-3 cells with IL-1β and hIL-1Ra (WT and mutant) (**Figure 4A**). Treatment with six hIL-1Ra mutants, E127N, E127S, E127K, E127Q, E127H and E127V, resulted in significant reductions in IL-1β mRNA expression compared with that produced by iPT hIL-1Ra WT as well as hIL-1Ra WT obtained from R&D Systems (RD hIL-1Ra WT) (**Figure S7** and **S8**). Specifically, five of the six hIL-1Ra variants reduced IL-1β mRNA expression by 43-48% compared with RD hIL-1Ra WT, with one (E127K) reducing IL-1β mRNA expression by a lesser amount (35%). Similar results were obtained using iPT hIL-1Ra WT as a control, with reductions of 41-45% observed for E127N, E127S, E127Q, E127H and E127V, and 32% for E127K. These data demonstrate that all six hIL-1Ra mutants exhibited superior anti-inflammatory efficacy compared with that of iPT hIL-1Ra WT and commercially obtained RD hIL-1Ra WT.

A further analysis of the anti-inflammatory actions of WT and mutant hIL-1Ra investigating expression of mRNA for the inflammatory cytokine, IL-6 (**Figure 4B**), revealed that treatment with E127N, E127S, E127K, E127Q, E127H, or E127V also significantly reduced IL-6 mRNA expression compared with iPT hIL-1Ra WT, as well as RD hIL-1Ra WT. Notably, the E127Q mutant showed the greatest reduction in IL-6 mRNA expression relative to the other variants, reducing IL-6 expression by 53% and 49% compared with RD hIL-1Ra WT and iPT hIL-1Ra WT, respectively. By comparison, the other mutants showed reductions ranging from 33% to 42% compared with RD hIL-1Ra WT and 25% to 35% compared with iPT hIL-1Ra WT. However, immunoblot analyses of NF-κB phosphorylation and IκBα degradation revealed that there was no appreciable difference between WT and E127Q in these parameters (**Figure S9**), likely reflecting the presence of a ceiling effect and the limited sensitivity of the assays under near-saturating inhibitory conditions. These results confirm that the six hIL-1Ra mutants demonstrate superior anti-inflammatory efficacy compared with our hIL-1Ra WT and commercial hIL-1Ra WT, with E127Q being particularly potent in reducing both IL-1β and IL-6 mRNA expression.

### The hIL-1Ra E127Q mutant potently blocks IL-1β-induced NMDAR hyperactivation under neuroinflammation

**Figure 5:**
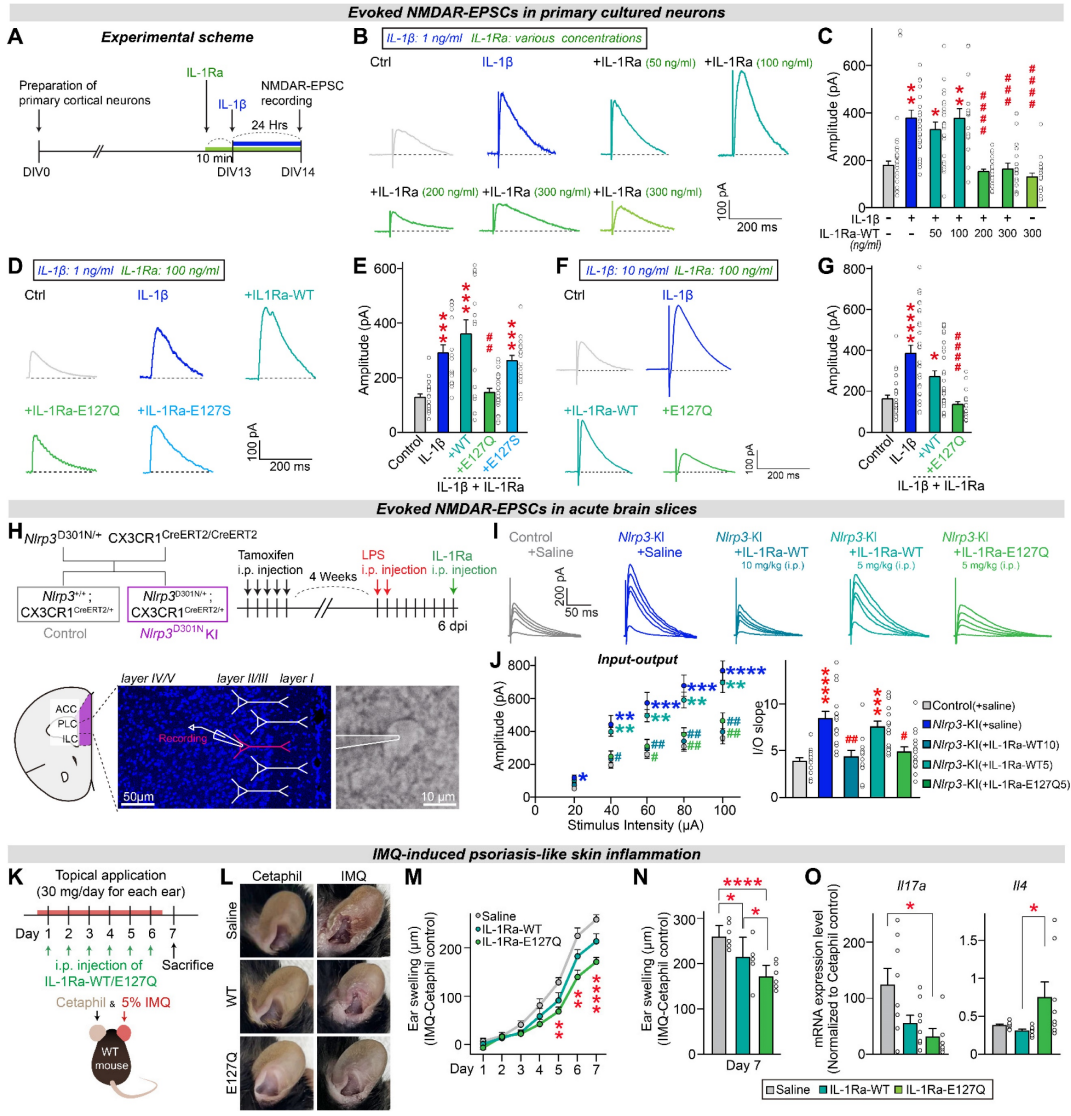
** hIL-1Ra E127Q mutant more potently blocks the effects of IL-1β on NMDAR transmission than hIL-1Ra WT.** (**A**) Schematic illustration of the experimental protocol. (**B** and** C**) Illustrative traces (**B**) and quantification (**C**) of evoked NMDAR-EPSCs measured in primary neurons exposed to vehicle, hIL-1Ra alone (300 ng/ml) or IL-1β (1 ng/ml), with or without pretreatment with hIL-1Ra (50, 100, 200 or 300 ng/ml). Data are presented as means ± SEMs (N values/group: Ctrl = 23; IL-1β = 27; +IL-1Ra 50 = 23; +IL-1Ra 100 = 16; +IL-1Ra 200 = 27; +IL-1Ra 300 = 17; IL-1Ra 300 alone = 21; *p < 0.05, **p < 0.01 vs. Veh; ^###^*p* < 0.001, ^####^*p* < 0.0001 vs. IL-1β; Kruskal-Wallis test). (**D** and** E**) Illustrative traces (**D**) and quantification (**E**) of evoked NMDAR-EPSCs measured in primary neurons exposed to vehicle or IL-1β (1 ng/ml), with or without pretreatment with WT or mutant hIL-1Ra (100 ng/ml). Data are presented as means ± SEMs (N values/group: Ctrl = 21; IL-1β = 18; +IL-1Ra-WT = 16; +IL-1Ra E127Q = 23; +IL-1Ra E127S = 19; ***p < 0.001 vs. Veh; ^##^*p* < 0.01 vs. IL-1β; Kruskal-Wallis test). (**F** and** G**) Illustrative traces (**F**) and quantification (**G**) of evoked NMDAR-EPSCs measured in primary neurons exposed to vehicle or IL-1β (10 ng/ml), with or without pretreatment with WT or mutant hIL-1Ra (100 ng/ml). Data are presented as means ± SEMs (N values/group: Ctrl = 28; IL-1β = 28; +IL-1Ra-WT = 30; +IL-1Ra E127Q = 21; *p < 0.05, ****p < 0.0001 vs. Veh; ^####^*p* < 0.0001 vs. IL-1β; Kruskal-Wallis test). (**H**) Overview of the breeding scheme and experimental design. (**I**) Evoked NMDAR-EPSC traces measured from Ctrl or *Nlrp3*^D301N^ KI mice injected with LPS, with or without acute hIL-1Ra WT or E127Q administration at 6 dpi. (**J**) Quantification of evoked NMDAR-EPSC amplitudes and fitted linear I-O slopes. Data are presented as means ± SEMs (N values/group: Control = 15; *Nlrp3*^D301N^ KI = 15; *Nlrp3*^D301N^ KI + IL-1Ra-WT10 = 12; *Nlrp3*^D301N^ KI + IL-1Ra-WT5 = 15; *Nlrp3*^D301N^ KI + IL-1Ra-Q5 = 16; ****p < 0.0001, ***p < 0.001 vs. Control; ^#^*p* < 0.05, ^##^*p* < 0.01 vs. *Nlrp3*^D301N^ KI; Kruskal-Wallis test). (**K**) Schematic illustration of the experimental protocol. Mice were topically treated once daily for 6 consecutive days with imiquimod (IMQ) cream or Cetaphil (control) on each ear. In parallel, i.p. injections of saline, WT IL-1Ra, or E127Q IL-1Ra were administered once daily for 6 consecutive days. (**L**) Representative photographs of ears from each treatment group on day 7. (**M**) Time-course of ear swelling during the 7-day monitoring period, calculated as the difference in thickness between IMQ-treated and Cetaphil-treated ears of the same animal (n = 8/group; **p < 0.01, ****p < 0.0001; Two-way ANOVA with Tukey's multiple comparison). (**N**) Quantification of ear swelling at day 7 across groups. Data are presented as means ± SEMs (n = 8/group; *p < 0.05, ****p < 0.0001; One-way ANOVA with Tukey's multiple comparison). (**O**) qRT-PCR analysis of IL17A and IL-4 expression in the ears collected from mice i.p.-injected with saline, WT IL-1Ra, or E127Q IL-1Ra. Expression level of *Il17a* and *Il4* in IMQ-treated ears were normalized by expression level of *Il17a* and *Il4* in the corresponding Cetaphil ctrl ears. Data are presented as means ± SEMs (n = 6-8/group; *p < 0.05; Kruskal-Wallis test).

To further evaluate the efficacy of each hIL-1Ra mutant compared with hIL-1Ra WT, we tested their effects on physiological properties of neurons using a cultured neuron system. We previously found that IL-1β treatment increases the amplitudes of NMDAR (N-methyl-D-aspartate receptor)-mediated evoked EPSCs (excitatory postsynaptic currents), without affecting those of AMPAR (α-amino-3-hydroxy-5-methyl-4-isoxazolepropionic acid receptor)-mediated EPSCs, by modulating the interaction between IL-1 receptors and NMDARs 25. Here, we first examined the concentration-dependence of hIL-1Ra WT inhibition of IL-1β-induced responses. To this end, we pretreated primary neurons with hIL-1Ra WT at concentrations ranging from 50 to 300 ng/ml; we then further treated them with 1 ng/ml IL-1β and measured the amplitudes of NMDAR-mediated EPSCs (**Figure 5A**). Electrophysiological analyses revealed that the IL-1β-induced increase in NMDAR-EPSC amplitude was normalized by pretreatment with hIL-1Ra at 200 or 300 ng/ml, but not by pretreatment with hIL-1Ra at 50 or 100 ng/ml (**Figure 5B-C**).

Next, we assessed the ability of 100 ng/ml of two selected hIL-1Ra mutants, E127S and E127Q, to inhibit IL-1β-induced responses. The IL-1β-induced increase in the amplitude of NMDAR-EPSCs was not normalized by pretreatment with 100 ng/ml of hIL-1Ra WT or the E127S mutant, but was completely suppressed by E127Q mutant at the same concentration (**Figure 5D-E**), demonstrating its superior inhibitory potency. To further address whether the superior efficacy of E127Q persists under conditions of more severe neuroinflammation, we repeated the same electrophysiological recordings using a higher concentration of IL-1β (10 ng/ml instead of 1 ng/ml). At this elevated dose, pretreatment with 100 ng/ml hIL-1Ra WT failed to normalize the IL-1β-induced increase in NMDAR-EPSC amplitudes, consistent with its limited potency. In contrast, the E127Q mutant still robustly suppressed the IL-1β-evoked enhancement of NMDAR responses, fully restoring amplitudes to baseline levels (**Figure 5F**-**G**). Together, these findings demonstrate that E127Q maintains its inhibitory activity even under conditions mimicking severe neuroinflammation, underscoring its markedly enhanced potency compared to hIL-1Ra WT.

Building on these *in vitro* observations, we next asked whether the superior efficacy of E127Q could be recapitulated *in vivo* using a genetic mouse model of excessive neuroinflammation. A previous study reported that excessive neuroinflammation in mice with microglia-specific knock-in (KI) of NLRP3 (NOD-like receptor protein 3) D301N, a point mutation identified in patients with autoinflammatory diseases, induced hyperactivation of NMDAR-mediated synaptic transmission in the medial prefrontal cortex (mPFC) 25. This hyperactivation was reversed by hIL-1Ra WT, indicating that IL-1β, as a downstream cytokine of the NLRP3 inflammasome, plays a key role in modulating NMDAR function 25. To compare the potency of hIL-1Ra WT and hIL-1Ra E127Q in blocking IL-1β action, we performed *ex vivo* electrophysiological recordings using *Nlrp3*^D301N^ KI mice. Acute treatment with 5 mg/kg of hIL-1Ra E127Q normalized the enhanced NMDAR-EPSCs in mPFC regions of adult *Nlrp3*^D301N^ KI mice, whereas the same dose of hIL-1Ra WT failed to produce a similar effect (**Figure 5H-J**). These results indicate that hIL-1Ra E127Q is more potent than hIL-1Ra WT in counteracting IL-1β-induced synaptic dysfunction under conditions of excessive neuroinflammation.

### The hIL-1Ra E127Q mutant provides sustained protection against chronic inflammation

Having established the acute potency of the E127Q variant, we next examined its longitudinal efficacy of the E127Q variant. We employed an imiquimod (IMQ)-induced ear-inflammation model and monitored ear thickness daily for 7 days (**Figure 5K**-**L**). Mice received topical IMQ once per day, together with i.p. injections of saline, hIL-1Ra WT, or the E127Q variant. Throughout the experimental time course, E127Q treatment consistently suppressed ear swelling more effectively than saline, with a clear separation emerging from day 5 onward when inflammation was pronounced (**Figure 5M**). hIL-1Ra WT produced only a modest reduction compared to saline. At the day-7 endpoint, both WT and E127Q had significantly reduced swelling relative to saline, and the effect of E127Q was markedly greater than that of WT (**Figure 5N**). To further characterize local inflammatory responses, we quantified *Il17a* and *Il4* mRNA expression in ear tissues at the endpoint (**Figure 5O**). As reported in psoriasis models, IMQ treatment markedly upregulated *Il17a* expression and downregulated *Il4* expression 26, 27. Notably, E127Q treatment substantially reduced the mRNA level of *Il17a* while elevating that of *Il4*, indicating restoration of an anti-inflammatory immune environment. These results demonstrate that E127Q provides superior *in vivo* anti-inflammatory efficacy during sustained inflammatory responses.

**Figure 6:**
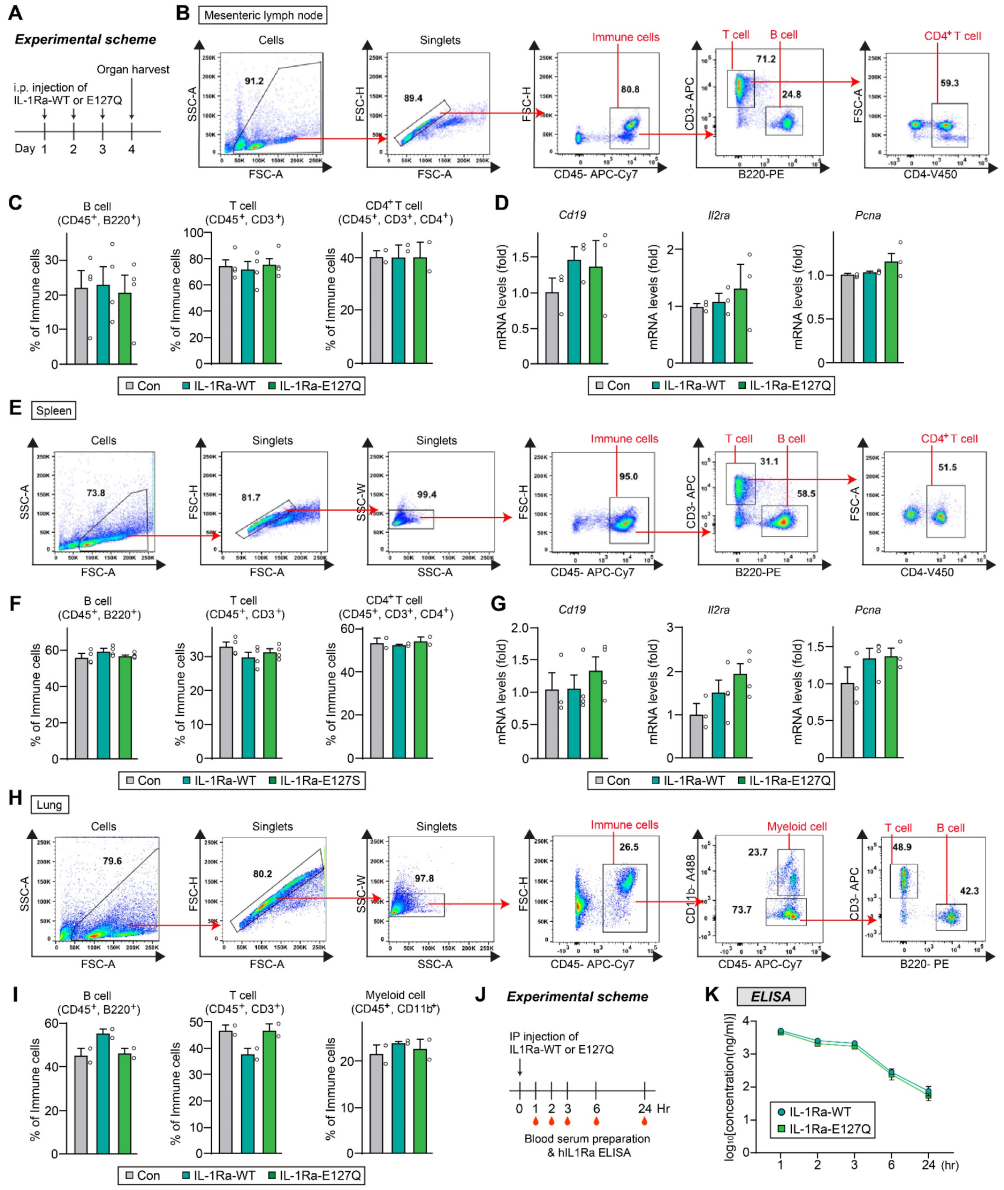
** Immunogenicity and *in vivo* stability of WT and E127Q IL-1Ra.** (**A**) Schematic illustration of the experimental protocol. (**B**-**D**) Lymph node analysis. (**B**) Representative flow cytometry plots showing the gating strategies used for B cells, T cells, and CD4⁺ T cells. (**C**) Quantification of B cell (CD45^+^, B220^+^), T cell (CD45^+^, CD3^+^) and CD4⁺ T cell (CD45^+^, CD3^+^, CD4^+^) populations in lymph node tissues from mice injected with saline (control), WT IL-1Ra, or E127Q IL-1Ra, as assessed using flow cytometric analysis (B cells and T cells, n = 4; CD4^+^ T cells, n = 2). (**D**) Relative mRNA levels of *Cd19*, *Il2ra*, and *Pcna* in lymph node extracts (n = 3). (**E**-**G**) Spleen analysis. (**E**) Representative flow cytometry plots of B cells, T cells, and CD4⁺ T cells. (**F**) Quantification of splenic immune cell populations (B cells and T cells, n = 4; CD4^+^ T cells, n = 2). (**G**) Relative mRNA levels of *Cd19*, *Il2ra*, and *Pcna* in spleen extracts (n = 3-4). (**H** and **I**) Lung analysis. (**H**) Representative flow cytometry plots of B cells (CD45⁺B220⁺), T cells (CD45⁺CD3⁺), and myeloid cells (CD45⁺CD11b⁺). (**I**) Quantification of lung immune cell populations (n = 2). (**J**) Schematic illustration of the experimental design. hIL-1Ra WT or E127Q was administered intraperitoneally into mice, and blood samples were collected at the indicated time points. (**K**) Serum concentrations of hIL-1Ra WT and E127Q, as determined by ELISA and plotted on a log_10_ scale. Data are presented as means ± SEMs (n = 4/group).

To further address immunological safety, mice were administered hIL-1Ra WT or E127Q intraperitoneally once daily for 3 consecutive days, and immune responses were assessed 24 h after the final injection. Flow cytometric analyses of mesenteric lymph nodes, spleen, and lung showed that the frequencies of B cells, total T cells, and CD4⁺ T cells were comparable between groups (**Figure 6**). In addition, quantitative RT-PCR analyses of spleen and lymph node tissues revealed that there were no differences in the mRNA expression levels of *Cd19*, *Il2ra*, or *Pcna* (**Figure 6D** and **G**). These findings demonstrate that repeated administration of E127Q does not induce detectable immune activation, supporting its immunological safety *in vivo*. Additionally, ELISA of serum from i.p.-injected mice revealed that hIL-1Ra WT and the E127Q variant exhibited highly comparable pharmacokinetic profiles, indicating that their *in vivo* stabilities are similar (**Figure 6J-K**).

## Discussion

In the current study, we implemented a structure-based design approach to engineer hIL-1Ra variants with enhanced anti-inflammatory properties, marking a significant advancement in the development of IL-1 inhibitors. Our *in silico* mutagenesis efforts, informed by molecular dynamics simulations that highlight the pivotal role of the E127 residue of hIL-1Ra in the stability of the hIL-1Ra/IL-1R1 complex, led to the identification of six variants predicted to exhibit substantial increases in binding affinity (ΔΔG of -7.8 + -0.9 kcal/mol compared with hIL-1Ra WT). *In vitro* evaluations confirmed our computational predictions, demonstrating that these six variants exhibited improved anti-inflammatory activity. Notably, the E127Q and E127S variants significantly reduced mRNA levels of the key pro-inflammatory cytokines IL-1β and IL-6-central players in the pathogenesis of diseases such as rheumatoid arthritis, gout, and other IL-1-driven conditions. Remarkably, these novel hIL-1Ra variants exhibited up to a 53% improvement in anti-inflammatory efficacy compared with hIL-1Ra WT prepared by us (iPT hIL-1Ra WT) or obtained commercially (RD hIL-1Ra WT), emphasizing their enhanced ability to suppress cytokine expression. The E127Q variant was particularly effective, exhibiting the highest efficacy in inhibiting IL-6 expression, highlighting its potential as a more effective therapeutic agent against a broad range of inflammatory disorders. We further investigated the effects of hIL-1Ra variants on neuronal physiology using cultured neurons, finding that hIL-1Ra E127Q effectively blocked IL-1β-induced disruptions in NMDAR-mediated excitatory synaptic transmission more potently (i.e., at a lower concentration) than hIL-1Ra WT. Building on these findings, we performed electrophysiological recordings in the *Nlrp3*^D301N^ KI mouse model, which recapitulates features of excessive neuroinflammation. Acute administration of hIL-1Ra E127Q normalized the heightened NMDAR-EPSCs in the mPFC, a key brain region implicated in neuroinflammatory and neuropsychiatric disorders, whereas hIL-1Ra WT, administered at the same concentration, did not, underscoring the superior efficacy of the E127Q variant. The normalization of NMDAR-mediated synaptic transmission is particularly relevant, given that dysregulation of glutamatergic signaling has been implicated in the pathophysiology of various neuroinflammatory conditions. Importantly, the use of the *Nlrp3*^D301N^ KI mouse model allowed us to evaluate the therapeutic potential of hIL-1Ra variants in a setting that closely mimics pathological neuroinflammation driven by inflammasome hyperactivation. The ability of hIL-1Ra E127Q to reverse synaptic abnormalities in this model highlights its promise as a candidate for intervention in neuroinflammatory diseases where IL-1β signaling is dysregulated. While these results collectively demonstrated that the engineered hIL-1Ra variants exhibit robust anti-inflammatory efficacy across the tested neuronal and *in vivo* systems, we acknowledge that validation in primary human immune-relevant cells (e.g., microglia, monocytes, and/or macrophages) remains an important future step to further establish the generalizability of our findings.

These bio-better hIL-1Ra variants represent a significant step forward compared with current treatment options. The alignment between our computational predictions and experimental validations across cellular systems and animal model findings provides a solid foundation for the rational design of next-generation biologics aimed at chronic inflammatory diseases. Our future research will focus on optimizing variants' *in vivo* efficacy, stability, and overall therapeutic benefits. Specifically, we are investigating the application of long-acting platforms, such as fusion with albumin 28, to extend the plasma half-life of these promising variants. This approach aims to reduce the frequency of administration, improve patient compliance, and potentially enhance sustained therapeutic benefits in patients with chronic inflammatory diseases*.*

## Supplementary Material

Supplementary figures and methods.

## Figures and Tables

**Figure 3:**
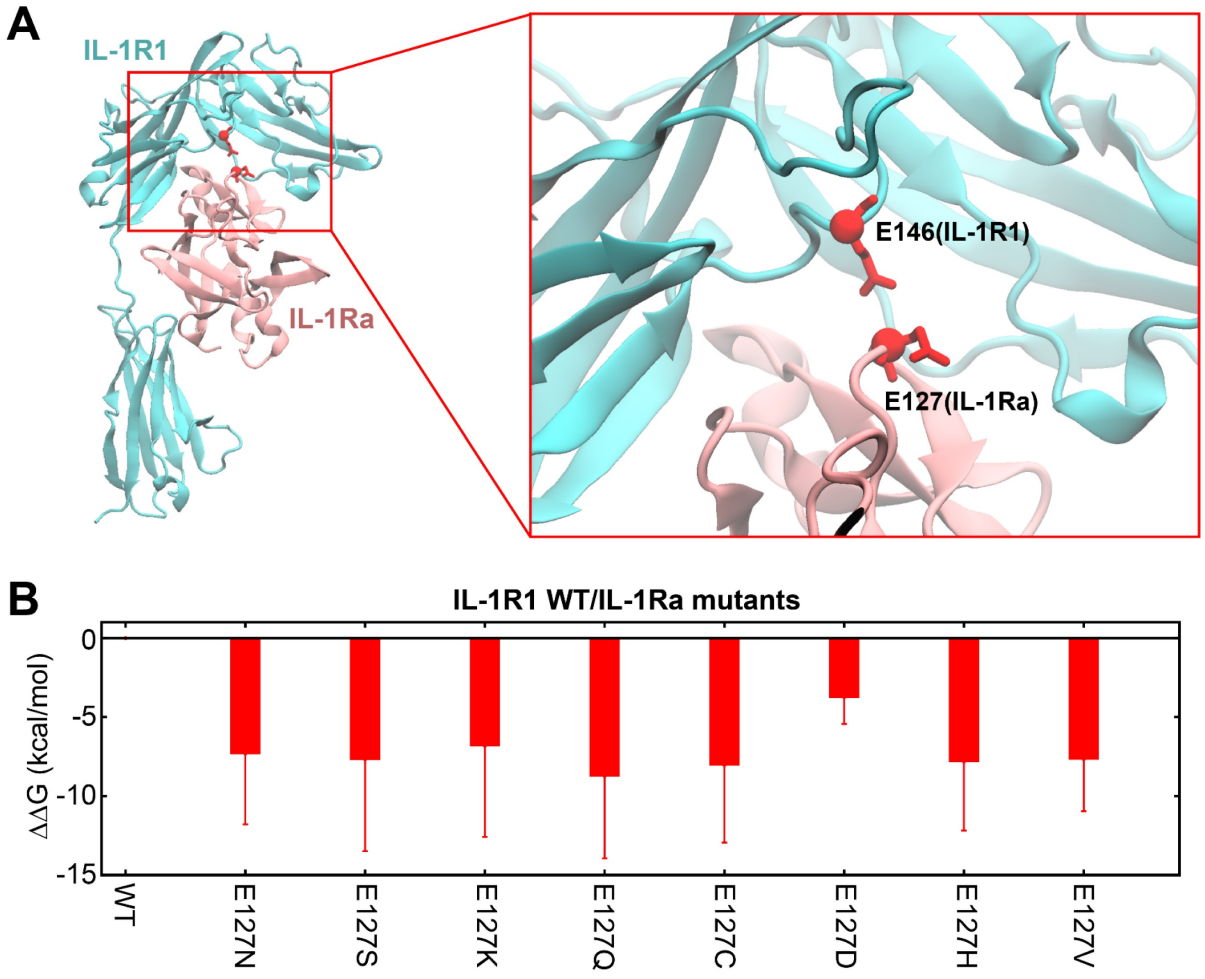
** Structural context of E127 in hIL-1Ra and predicted binding free energy changes of its variants.** (**A**) 3D structure of the IL-1R1 (cyan) and hIL-1Ra (pink) complex. The E127 residue of hIL-1Ra is highlighted, showing its close proximity to E146 of IL-1R1 (red sphere and stick). This proximity underlies the strong repulsive electrostatic interactions that are modulated by mutations involving E127. (**B**) Calculated binding free energy differences (ΔΔG) for eight single-point E127 mutants of hIL-1Ra compared with hIL-1Ra WT. The eight variants (E127N, E127S, E127K, E127Q, E127C, E127D, E127H, E127V) displayed favorable (negative) ΔΔG values.

## Abbreviations

AMPAR: α-amino-3-hydroxy-5-methyl-4-isoxazolepropionic acid receptor
CAPS: cryopyrin-associated periodic syndrome
EPSC: excitatory postsynaptic current
IL-1: interleukin-1
IL-1Ra: interleukin-1 receptor antagonist
IL-1R1: type I interleukin-1 receptor
IMQ: imiquimod
KI: knock-in
LPS: lipopolysaccharide
MAPK: mitogen-activated protein kinase
mPFC: medial prefrontal cortex
NF-κB: nuclear factor-κB
NLRP3: NOD-like receptor protein 3
NMDAR: N-methyl-D-aspartate receptor
RA: rheumatoid arthritis
TI-MD: thermodynamic integration-molecular dynamics
