# Supplementary material for: Development of New IL-1R Antagonists with Improved Anti-inflammatory Efficacy
Source: Theranostics. 2026 Jan 1;16(5):2561–75. doi: 10.7150/thno.120259 (PMC12712897; doi:10.7150/thno.120259)
Supplement: Supplementary file 1 — Supplementary figures and methods. [file thnov16p2561s1.pdf]

Supplementary Materials for

Development of new IL-1R antagonists with improved anti-inflammatory efficacy

Mooseok Kang[1,*], Ae-Ree Lee[1,*], Hyeji Jung[2,3,*], Gyubin Jang[2,3], Byeongchan Kim[2], Sung-Hyun Yoon[4], Je-Wook Yu[4], Jaewon Ko[2,3], Ji Won Um[2,3,$] and Iksoo Chang[1,$]

[1] Cytokine Innovation Center, iProtein Therapeutics Co. Ltd., Industry-University Cooperation Building R7-208, 333 Techno Jungangdae-Ro, Hyeonpoong-Eup, Dalseong-Gun, Daegu 42988, Korea

[2] Department of Brain Sciences, Daegu Gyeongbook Institute of Science and Technology (DGIST), 333 Techno Jungangdae-Ro, Hyeonpoong-Eup, Dalseong-Gun, Daegu 42988, Korea

[3] Center for Synapse Diversity and Specificity, Daegu Gyeongbook Institute of Science and Technology(DGIST), 333 Techno Jungangdae-Ro, Hyeonpoong-Eup, Dalseong-Gun, Daegu 42988, Korea

[4]Department of Microbiology and Immunology, Institute for Immunology and Immunological Diseases, Brain Korea 21 PLUS Project for Medical Science, Yonsei University College of Medicine, Seoul 03722, Korea

*These authors contributed equally

[$]Correspondence: Ji Won Um (jiwonum@dgist.ac.kr) or Iksoo Chang (ikspro@iprothera.com)

This file includes:

Supplementary figure S1–S9

Supplementary methods

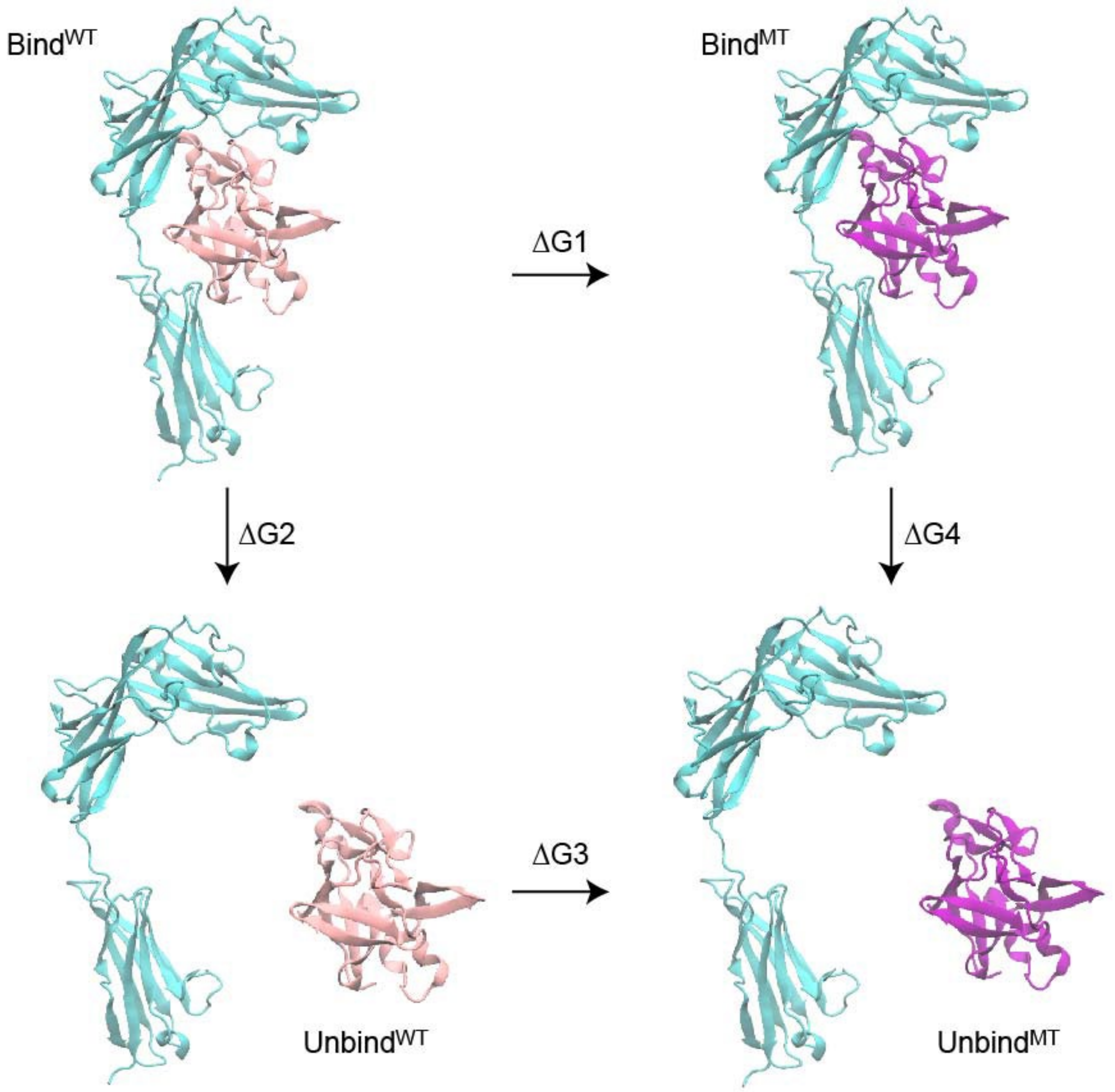

Supplementary Figure S1. Thermodynamic cycle for calculating the binding free energy difference (ΔΔG) between wild-type and mutant hIL-1Ra

The bound and unbound structures of the IL-1R1/hIL-1Ra complex are shown. Using the thermodynamic cycle, the binding free energy difference (ΔΔG) = ΔG2 – ΔG4 was calculated by determining ΔG1 – ΔG3.

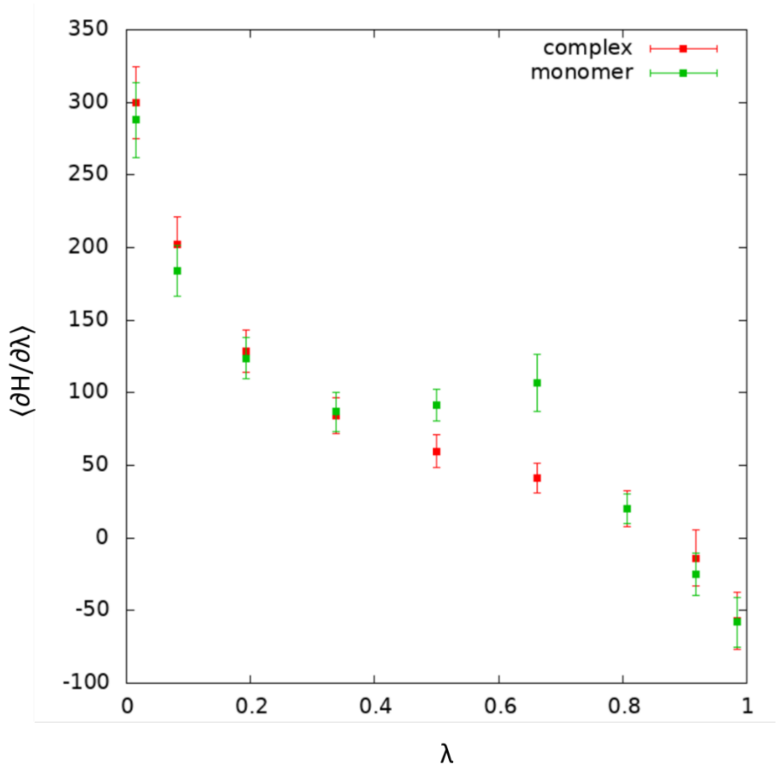

Supplementary Figure S2. ⟨∂H/∂λ⟩ vs. λ profiles for E127Q TI calculations. The average derivatives of the Hamiltonian with respect to the coupling parameter (⟨∂H/∂λ⟩) are plotted against λ for both the complex (red squares) and monomer (green squares) systems. Error bars represent the SEM, indicating the precision of the sampled values at each λ window. The smooth nature of these profiles indicates that the sampling was sufficient for accurate integration.

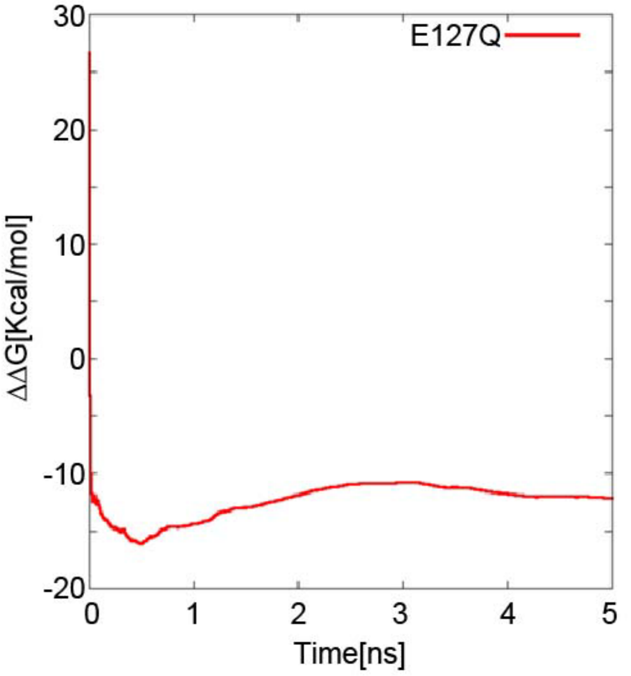

Supplementary Figure S3. Convergence behavior of ΔΔG for E127Q. The cumulative ΔΔG value is plotted as a function of simulation time. The graph demonstrates that the calculation reaches a stable plateau after the initial equilibration, indicating that the free energy calculation converges.

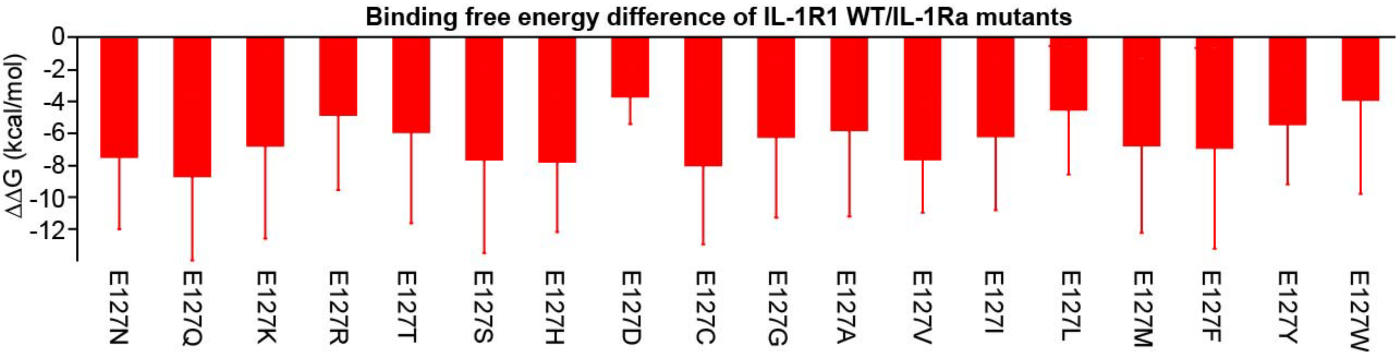

Supplementary Figure S4. Binding free energy differences (ΔΔG) for the 18 single-point mutants at the E127 position in hIL-1Ra.

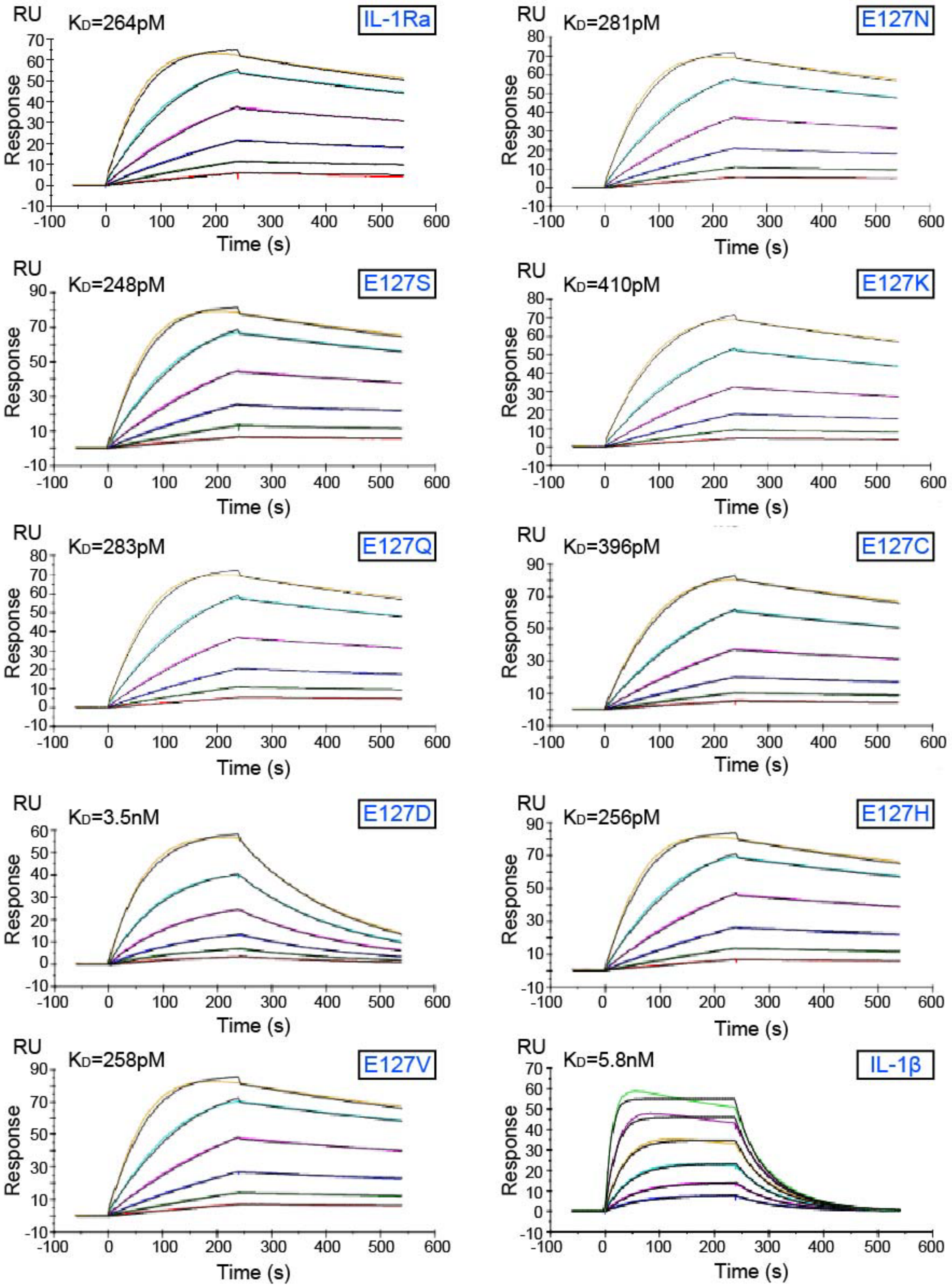

Supplementary Figure S5. Surface plasmon resonance (SPR) analysis of IL-1R1 binding to hIL-1Ra WT, its E127 mutants, and IL-1β. Representative SPR sensorgrams illustrate the binding of hIL-1Ra WT, its E127 mutants, and IL-1β to immobilized IL-1R1. The derived equilibrium dissociation constant (K_D) values are indicated for each protein. Overall, most of the hIL-1Ra variants demonstrate significantly stronger binding than IL-1β to IL-1R1. They also exhibit K_D values similar to those of WT hIL-1Ra, indicating that the direct binding affinities.

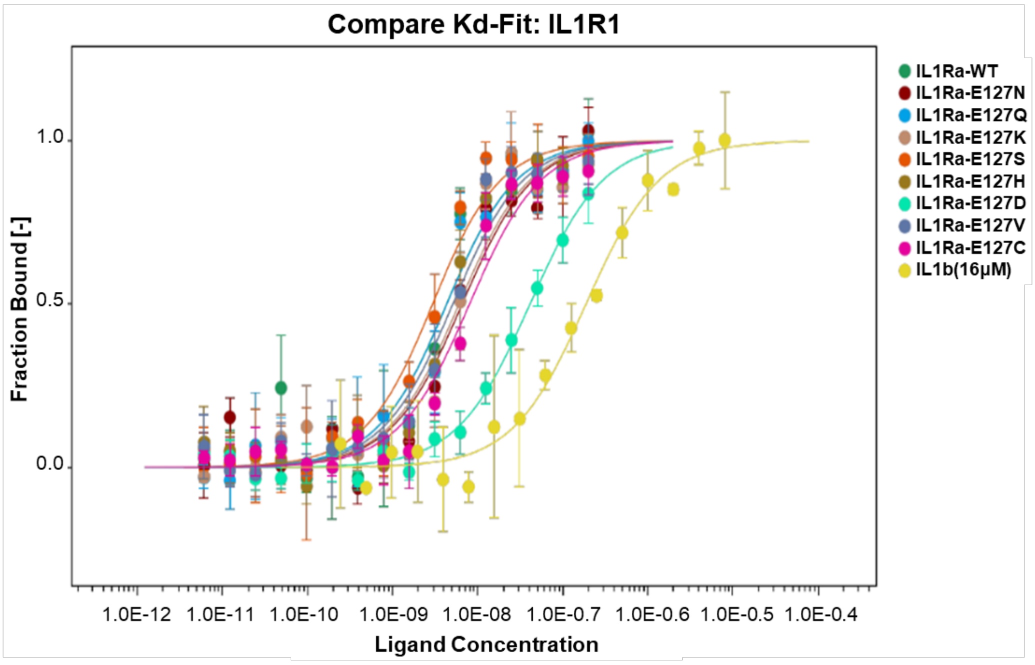

Supplementary Figure S6. Microscale thermophoresis (MST) analysis of the IL-1R1 binding affinity for hIL-1Ra WT, its E127 mutants, and IL-1β. Normalized fluorescence curves are presented, demonstrating the binding of IL-1R1 to increasing concentrations of hIL-1Ra WT, its E127 mutants, and IL-1β. The observed shift in thermophoresis reflects the molecular binding events. The derived equilibrium dissociation constant (K_D) for each interaction is indicated on the respective curve.

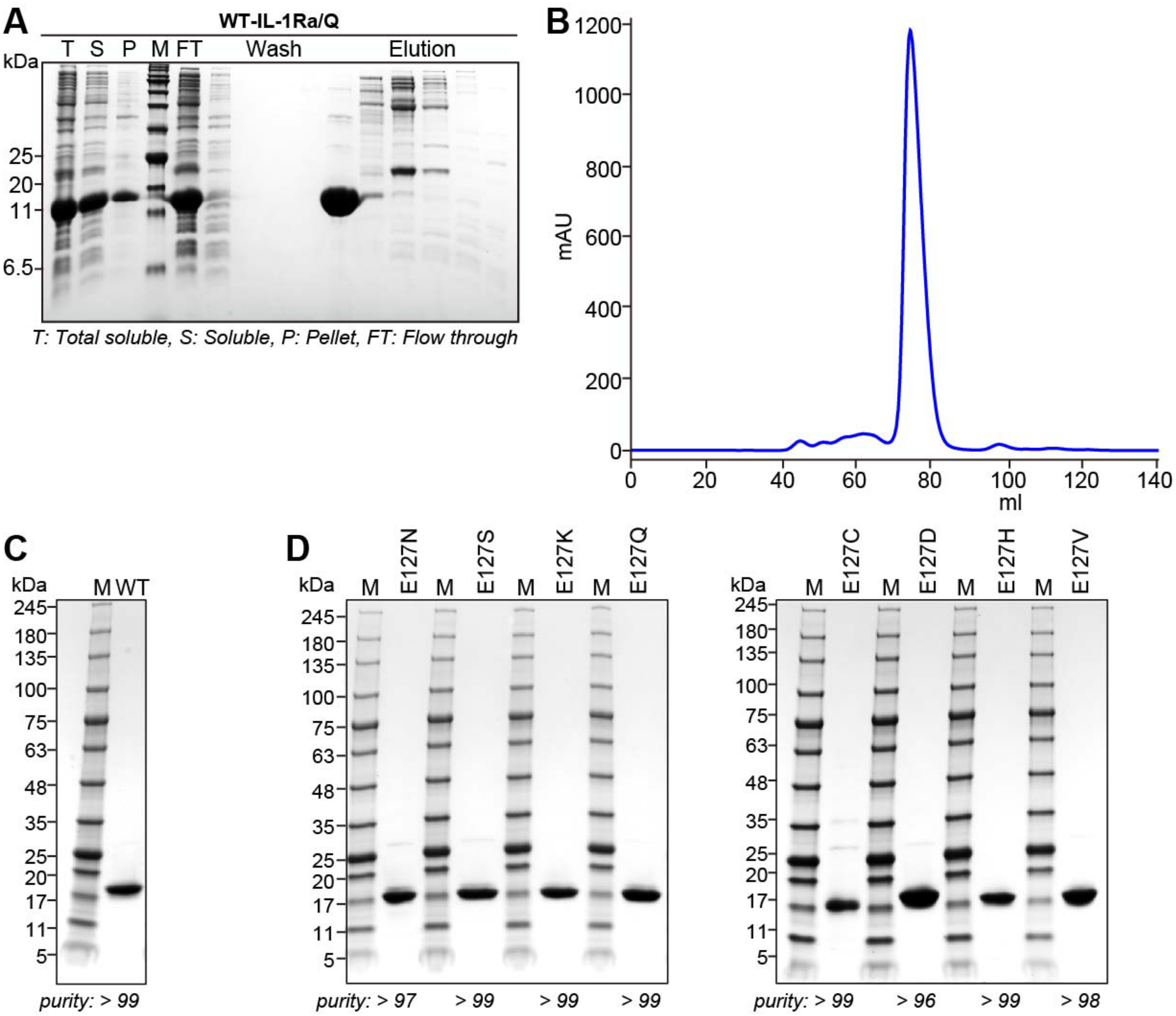

Supplementary Figure S7. Size exclusion chromatography (SEC) profiles, final purity, and production yields of wild-type hIL-1Ra and its variants.

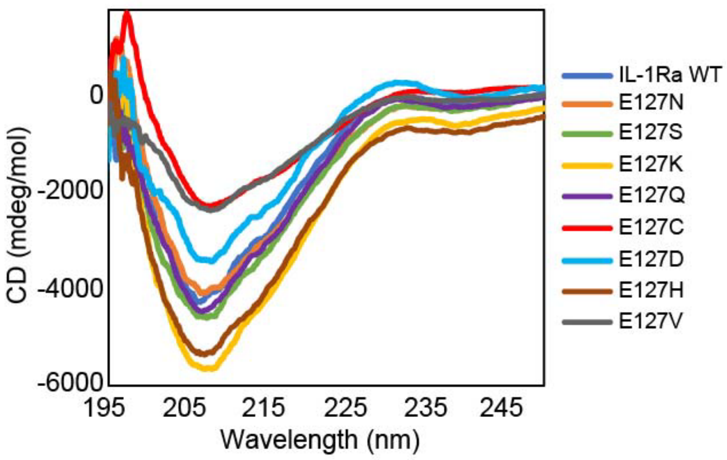

Supplementary Figure S8. Circular dichroism (CD) spectra of wild-type hIL-1Ra and its variants.

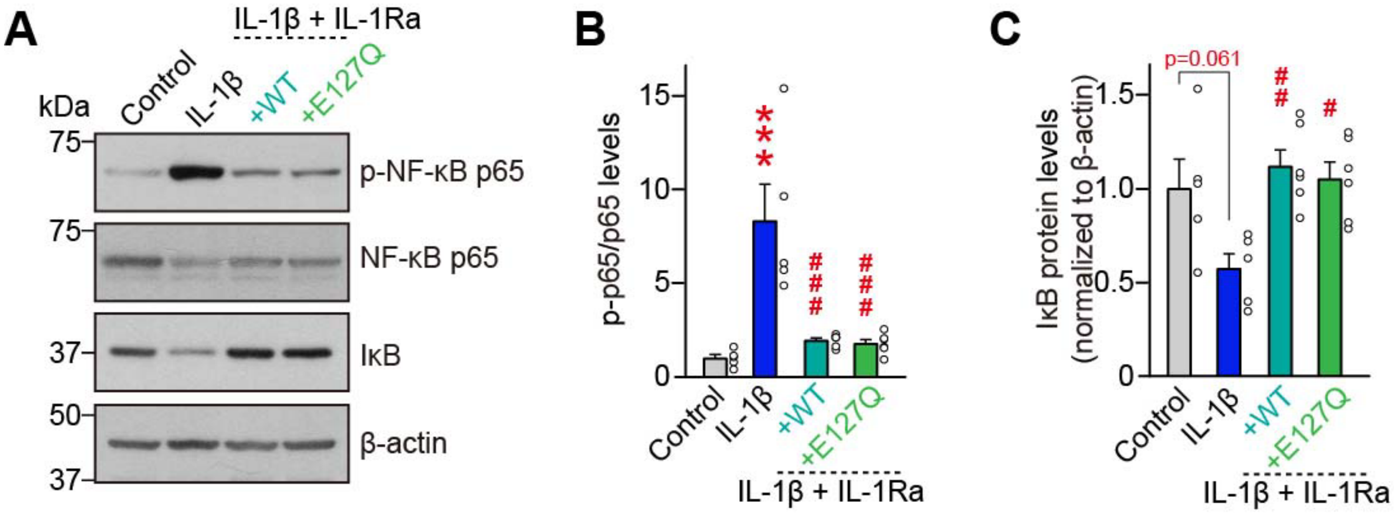

Supplementary Figure S9. Both hIL-1Ra WT and E127Q fully inhibit IL-1β-induced NF-κB activation in human microglial cells.

(**a**) Representative immunoblots of phosphorylated NF-κB p65 (p-p65), total NF-κB p65, and IκBα in HMC3 human microglial cells treated with IL-1β (10 ng/ml) in the presence or absence of hIL-1Ra WT or E127Q (50 ng/ml) for 20 min. β-actin served as a loading control.

(**b**) Quantification of p-p65 normalized to total p65. Data are presented as means ± SEMs (n=5-6 independent experiments; $^{***}p < 0.001$ vs. Control; $^{\#\#\#}p < 0.001$ vs. IL-1β; One-way ANOVA with Tukey's multiple comparison).

(**c**) Quantification of IκBα protein levels. Data are presented as mean ± SEMs. Data are presented as means ± SEMs (n = 5–6 independent experiments; $^{\#}p < 0.05$, $^{\#\#}p < 0.01$ vs. IL-1β; One-way ANOVA with Tukey's multiple comparison).

Supplementary methods

Thermodynamic integration (TI) calculation with softcore potentials

The binding free energy differences were calculated using a thermodynamic cycle (**Fig. S1**). ΔG2 (ΔG4) represents the binding free energy between IL-1R1/hIL-1Ra of the wild-type hIL-1Ra (or mutant); ΔG1 (ΔG3) represents the free energy difference between WT and mutant hIL-1Ra complex (or monomer). The thermodynamic equality is ΔG2 + ΔG3 – ΔG4 – ΔG1 = 0. To obtain the binding free energy differences between WT and mutants, the equation for the thermodynamic cycle can be rearranged as ΔG2 – ΔG4 = ΔG1 – ΔG3 = ΔΔG.

The TI method, using a mixed potential function, was applied to calculate ΔG1 and ΔG3, while softcore potentials were used to smooth the appearance and disappearance of atoms in van der Waals (vdW) and electrostatic interactions in the hybrid state. The TI calculation employed the following Hamiltonian:

$$H_\lambda = H_{wt} + \lambda(H_{mut} - H_{wt}), \text{ (Eq. 1)}$$

where λ is the weight of the mutant state, varying from 0 to 1. For example, at λ = 0.4, the mixed state contains 60% of the WT structure and 40% of the mutant structure at the same position. This results in a final hybrid state with a mixed potential.

The equations for vdW and electrostatic potentials in softcore formulation are as follows:

$$V^{vdW}_{wt,disappearing} = 4\varepsilon(1-\lambda)\left[\frac{1}{\left[\alpha\lambda+\left(\frac{r_{ij}}{\sigma}\right)^6\right]^2} - \frac{1}{\alpha\lambda+\left(\frac{r_{ij}}{\sigma}\right)^6}\right] \text{ (Eq. 2)}$$

$$V^{vdW}_{mutant,appearing} = 4\varepsilon\lambda\left[\frac{1}{\left[\alpha(1-\lambda)+\left(\frac{r_{ij}}{\sigma}\right)^6\right]^2} - \frac{1}{\alpha(1-\lambda)+\left(\frac{r_{ij}}{\sigma}\right)^6}\right] \text{ (Eq.3)}$$

$$V^{elec}_{wt,disappearing} = (1-\lambda)\frac{q_i q_j}{4\pi\varepsilon\sqrt{\beta\lambda+r_{ij}^2}} \text{ (Eq. 4)}$$

$$V^{elec}_{mutant,appearing} = \lambda\frac{q_i q_j}{4\pi\varepsilon\sqrt{\beta(1-\lambda)+r_{ij}^2}} \text{ (Eq. 5)}$$

We constructed the coordinate and parameter files for the hybrid state using the tleap program, and modified the parameter files for mutation points in each chain using the parmed module in AMBER. Key input options for AMBER TI calculations include: icfe = 1 (free energy calculation), ifsc = 1 (softcore potential), scalpha = 0.5 (softcore potential parameter α), scbeta = 12.0 (softcore potential parameter β), temp0 = 300 (temperature in K), and clambda (λ).

The free energy difference (ΔG) was calculated using Gaussian quadrature, according to the formula:

$$\Delta G = \int_0^1 \frac{\partial G(\lambda)}{\partial\lambda}d\lambda = \int_0^1 \langle\frac{\partial V(\lambda)}{\partial\lambda}\rangle_\lambda \approx \sum_i w_i \langle\frac{\partial V(\lambda)}{\partial\lambda}\rangle_i \text{ (Eq. 6)}$$

To ensure the robustness and convergence of our TI calculations, a 9-point Gaussian quadrature was applied for the λ values: 0.01592, 0.08198, 0.19331, 0.33787, 0.5, 0.66213, 0.80669, 0.91802, and 0.98408, 0.98408, and weights (w_i) of 0.04064, 0.09032, 0.13031, 0.15617, 0.16512, 0.15617, 0.13031, 0.09032, and 0.04064, respectively. This non-uniform spacing ensured that the sampling was denser near the end states (λ=0 and λ=1), where free energy changes are often most pronounced. A 5-ns TI simulation was performed for each λ value, with 10 independent trajectories generated for each system to enhance sampling and statistical accuracy. The smoothness of the ⟨∂H/∂λ⟩ vs. λ profiles, along with associated error estimates, is presented in **Fig. S2**. The convergence behavior of the ΔΔG calculations over simulation time, which is shown in **Fig. S3**, demonstrates the stability and reliability of our results.

Binding affinity analysis of WT and mutant IL-1Ra by surface plasmon resonance (SPR) and microscale thermophoresis (MST)

To measure the binding force using the His Capture Kit, 100 mM N-hydroxysuccinimide (NHS) and 400 mM 1-ethyl-3-(3-dimethylaminopropyl) carbodimide (EDC) were mixed at a 1:1 ratio (v/v) and flowed for 420 seconds to activate each flow cell. The capture molecule was diluted with an immobilization buffer and flowed into the flow cell at a flow rate of 10 µl/min for 420 seconds at a flow rate of 9000–12000 RU. Then, 70 µl of 1M ethanolamine-HCl (pH 8.0) was flowed to deactivate the excess carboxyl group remaining on the CM5 chip surface. IL-1R1 was injected into the reference and sample flow cells at each cycle to capture them. An analyte was prepared by diluting the saturated response of analyte (IL1Ra wild type and mutant protein) at a maximum concentration in the section of 150 RU or less and diluting the corresponding concentration 5 to 6 times with 2 times serial dilution. Analytes of each concentration were sequentially injected by a multi-cycle analysis method and the combined sensorgram was generated. All utilized injection flow rates were adjusted by the BiacoreT200 control software. The binding force kinetics were calculated using a 1:1 binding evaluation model based on the BIA evaluation software manual. The reliability of the sensorgram fitting was evaluated as Chi2 and U-values, according to the manufacturer's manual provided with the His Capture Kit. Fluorescence labeling of 5 µM of IL-1R1 in 100 µL PBS buffer with 0.05% Tween-20 was performed using a Monolith Protein Labeling Kit RED-NHS 2nd Generation. Next, 10 µL of IL-1Ra WT and mutant proteins were prepared at 16 concentrations ranging from 400 nM to 12.2 pM using 1:1 serial dilution. Fluorescence-labeled IL-1R1 WT and variants (10 µL each) were mixed with 16 concentrations of ligands prepared by diluting 2 nM to prepare 1 nM of IL-1R1 with 200 nM to 6.104 pM of ligand mixture solution. IL-1β protein was prepared at 16 concentrations ranging from 16 µM to 0.488 nM using 1:1 serial dilution. Fluorescence-labeled IL-1R1 WT and variants (10 µL each) were mixed with 16 concentrations of ligands prepared by diluting 2 nM to prepare 1 nM of IL-1R1 with 8 µM to 0.244 nM of ligand mixture solution. MST was measured three times for each ligand using 20% excitation power and a medium MST power with a Monolith NT.115-pico machine. The average KD (dissociation constant) value was calculated three times using the Kdmodel of the MO affinity analysis program provided by Monolith.
